# Blood Metal Ion Thresholds to Identify Patients with Metal-on-Metal Hip Implants at Risk of Adverse Reactions to Metal Debris: An External Multicenter Validation Study of Birmingham Hip Resurfacing and Corail-Pinnacle Implants

**DOI:** 10.2106/JBJS.16.01568

**Published:** 2017-09-20

**Authors:** Gulraj S. Matharu, Fiona Berryman, Andrew Judge, Aleksi Reito, Jamie McConnell, Olli Lainiala, Stephen Young, Antti Eskelinen, Hemant G. Pandit, David W. Murray

**Affiliations:** 1 Nuffield Department of Orthopaedics, Rheumatology and Musculoskeletal Sciences, University of Oxford, Nuffield Orthopaedic Centre, Oxford, United Kingdom; 2 The Royal Orthopaedic Hospital, Birmingham, United Kingdom; 3 MRC Lifecourse Epidemiology Unit, Southampton General Hospital, University of Southampton, Southampton, United Kingdom; 4 Coxa Hospital for Joint Replacement, Tampere, Finland; 5 Warwick Hospital, Warwick, United Kingdom; 6 Leeds Institute of Rheumatic and Musculoskeletal Medicine (LIRMM), Chapel Allerton Hospital, Leeds, United Kingdom

## Abstract

**Background:**

The authors of recent studies have reported newly devised implant-specific blood metal ion thresholds to predict adverse reactions to metal debris (ARMD) in patients who have undergone unilateral or bilateral metal-on-metal (MoM) hip arthroplasty. These thresholds were most effective for identifying patients at low risk of ARMD. We investigated whether these newly devised blood metal ion thresholds could effectively identify patients at risk of ARMD after MoM hip arthroplasty in an external cohort of patients.

**Methods:**

We performed a validation study involving 803 MoM hip arthroplasties (323 unilateral Birmingham Hip Resurfacing [BHR], 93 bilateral BHR, and 294 unilateral Corail-Pinnacle implants) performed in 710 patients at 3 European centers. All patients underwent whole-blood metal ion sampling, and were divided into 2 groups: those with ARMD (leading to revision or identified on imaging; n = 75) and those without ARMD (n = 635). Previously devised implant-specific blood metal ion thresholds (2.15 μg/L of cobalt for unilateral BHR; 5.5 μg/L for the maximum of either cobalt or chromium for bilateral BHR; and 3.57 μg/L of cobalt for unilateral Corail-Pinnacle implants) were applied to the validation cohort, and receiver operating characteristic curve analysis was used to establish the discriminatory characteristics of each threshold.

**Results:**

The area under the curve, sensitivity, specificity, and positive and negative predictive values for the ability of each implant-specific threshold to distinguish between patients with and without ARMD were, respectively, 89.4% (95% confidence interval [CI] = 82.8% to 96.0%), 78.9%, 86.7%, 44.1%, and 96.9% for unilateral BHR; 89.2% (CI = 81.3% to 97.1%), 70.6%, 86.8%, 54.5%, and 93.0% for bilateral BHR; and 76.9% (CI = 63.9% to 90.0%), 65.0%, 85.4%, 24.5%, and 97.1% for unilateral Corail-Pinnacle implants. Using the implant-specific thresholds, we missed 20 patients with ARMD (2.8% of the patients in this series). We missed more patients with ARMD when we used the fixed thresholds proposed by regulatory authorities: 35 (4.9%) when we used the U.K. threshold of 7 μg/L for both cobalt and chromium (p = 0.0003), 21 (3.0%) when we used the U.S. threshold of 3 μg/L for both cobalt and chromium (p = 1.0), and 46 (6.5%) when we used the U.S. threshold of 10 μg/L for both cobalt and chromium (p < 0.0001).

**Conclusions:**

This external multicenter validation study confirmed that patients with blood metal ion levels below new implant-specific thresholds have a low risk of ARMD after MoM hip arthroplasty. Using these implant-specific thresholds, we missed fewer patients with ARMD compared with when the thresholds currently proposed by regulatory authorities were used. We therefore recommend using implant-specific blood metal ion thresholds when managing patients who have undergone MoM hip arthroplasty.

**Level of Evidence:**

Diagnostic Level III. See Instructions for Authors for a complete description of levels of evidence.

Large-diameter metal-on-metal (MoM) hip arthroplasties have had unexpected high failure rates, with many revisions performed for adverse reactions to metal debris (ARMD)^1-4^. In an attempt to identify ARMD early, worldwide regulatory authorities recommend regular patient follow-up^5-7^, including measurement of blood cobalt and chromium concentrations, which reflect in vivo bearing wear^8^. However, there is presently no international consensus on the blood metal ion threshold(s) that should cause clinical concern after a MoM hip arthroplasty, with previous studies showing that thresholds that have been used for identifying poorly functioning MoM hip replacements (3.5 to 7 μg/L) have had insufficient sensitivity^9-13^.

Two recent studies identified implant-specific blood metal ion thresholds in patients who had undergone unilateral or bilateral Birmingham Hip Resurfacing (BHR; Smith & Nephew) or unilateral Corail-Pinnacle total hip replacement (DePuy)^14,15^. These designs are 2 of the most commonly implanted MoM hip devices worldwide^3,16,17^. The newly devised implant-specific thresholds were most effective for identifying patients at low risk of ARMD. Application of fixed blood metal ion thresholds to all patients treated with MoM hip arthroplasty has been recommended by the U.K. Medicines and Healthcare products Regulatory Agency (MHRA) (7 μg/L for both cobalt and chromium)^5^ and by experts in the U.S. (3 μg/L and 10 μg/L for both cobalt and chromium)^18^. Investigators who used the new implant-specific thresholds^14,15^ missed fewer patients with ARMD than when they used the fixed thresholds proposed by regulatory authorities^5,18^. The implant-specific thresholds were derived in a large patient cohort (n = 783) and appear to be useful when managing patients with these particular MoM implant designs^14,15^. However, these implant-specific thresholds have to be externally validated in multiple centers before being used clinically^19^, even though this type of rigorous approach has not been used in many clinical prediction studies^20^.

We investigated whether these newly devised implant-specific blood metal ion thresholds could effectively identify MoM-treated patients at risk of ARMD in an external cohort of patients with BHR and Corail-Pinnacle implants.

## Materials and Methods

An external multicenter validation cohort study was performed in 3 European arthroplasty institutions to investigate the ability of the previously devised implant-specific thresholds to discriminate between patients with and without ARMD^21^. BHRs and/or 36-mm MoM Corail-Pinnacle total hip replacements had been implanted at these centers; specific information about these implant designs was described previously^14^. Ethical approval was not required for our study as all centers prospectively followed patients with MoM implants according to published regulatory guidance^5,7^. The BHR and Corail-Pinnacle patient cohorts at each center have been previously described, with the follow-up protocols and blood metal ion analytical methods also detailed^22-26^. Specific details related to each center are provided below.

### Center 1

Between 1999 and 2009, 646 BHR implants were used^22^. Following arthroplasty, all patients underwent clinical assessment, which included a history, examination, anteroposterior pelvic radiographs, and completion of the Oxford Hip Score (OHS) questionnaire^27^. Patients with hip problems (pain, swelling, or noises) and/or a suboptimal OHS (≤41 of 48 points) underwent blood metal ion sampling and hip ultrasound examination^4^. Asymptomatic patients with a BHR also underwent blood sampling and ultrasound if they were considered to have risk factors for ARMD (a small femoral component, a malpositioned acetabular component, or radiographic abnormalities including osteolysis or neck narrowing)^28-32^ or if they had concerns about the implant because of media attention. In addition, 128 asymptomatic patients with a BHR (OHS > 41 points) underwent blood sampling and ultrasound as part of a previous study^23^. Metal artifact reduction sequence magnetic resonance imaging (MARS-MRI) was reserved for equivocal or complex cases^33^.

### Center 2

At this center, 425 BHR implants were used^24^ between 2001 and 2012 and 17 Corail-Pinnacle implants were used between 2005 and 2010^25^. Following arthroplasty, all patients, regardless of symptoms, underwent clinical assessment including pelvic and hip radiographs and blood metal ion sampling. All symptomatic patients, and asymptomatic patients with blood cobalt and/or chromium concentrations of ≥5 μg/L, underwent MARS-MRI or, if that was contraindicated, ultrasound. Selected asymptomatic patients with blood metal ion concentrations of <5 μg/L also underwent cross-sectional imaging at the surgeons’ discretion—for example, if there were concerns about the radiographic appearance or if the contralateral arthroplasty required cross-sectional imaging.

### Center 3

Between 2006 and 2011, 601 Corail-Pinnacle implants were performed^26^. Following arthroplasty, all patients, regardless of symptoms, underwent clinical assessment including pelvic radiographs and blood metal ion sampling. All symptomatic patients, and asymptomatic patients with blood cobalt and/or chromium concentrations of >7 μg/L (the MHRA upper limit)^5^, underwent ultrasound examination. MARS-MRI was reserved for equivocal or complex cases. Selected asymptomatic patients with blood metal ion concentrations of ≤7 μg/L also underwent cross-sectional imaging at the surgeons’ discretion as described above.

### Inclusion/Exclusion Criteria and Definitions

**Fig. 1:**
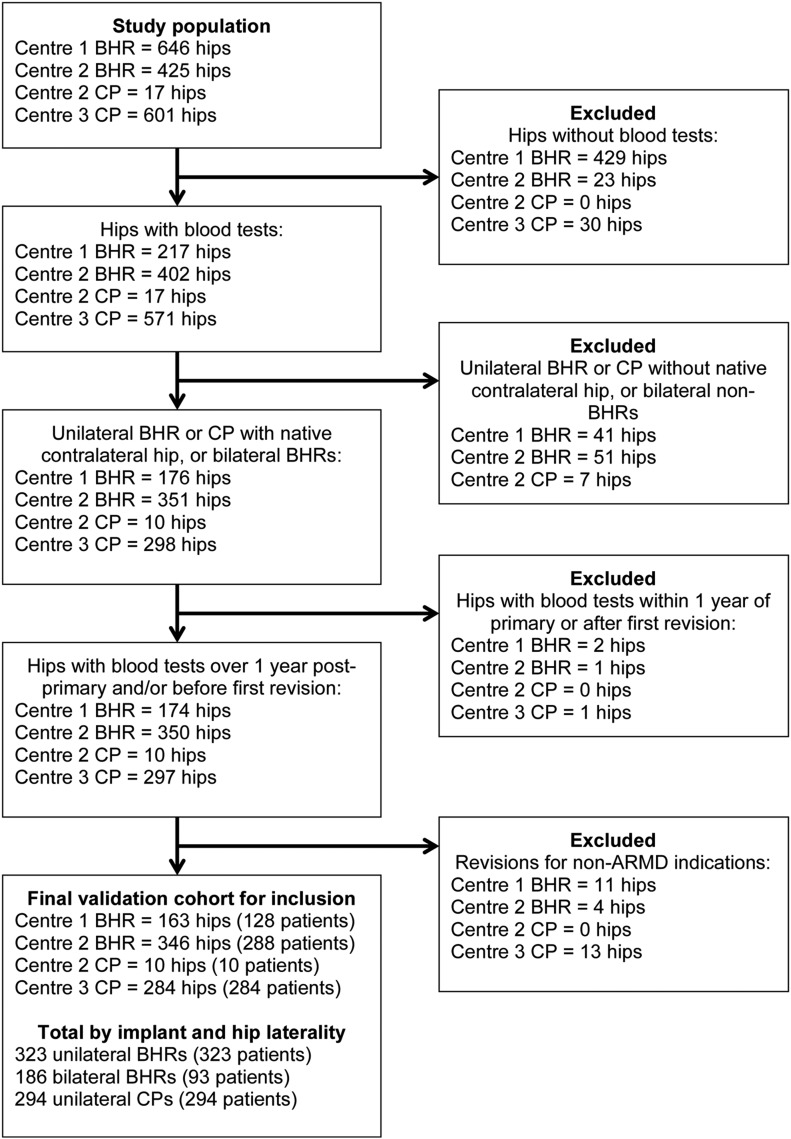
Study inclusion and exclusion criteria. ARMD = adverse reactions to metal debris, BHR = Birmingham Hip Resurfacing, and CP = Corail-Pinnacle.

The same selection criteria (Fig. 1) and definitions used by the investigators who derived the blood metal ion thresholds^14,15^ were applied to this validation cohort. For the present study, whole-blood cobalt and chromium samples obtained up until October 2016 were eligible for inclusion. Patients with a unilateral arthroplasty had a native contralateral hip at the time of blood sampling, whereas those who had undergone bilateral arthroplasty were included in the present study only if both implants were a BHR at the time of sampling. All blood samples were obtained at least 1 year following the primary arthroplasty and before any revision surgery. Patients who had undergone blood sampling before revision for non-ARMD indications had been excluded from the previous studies^14,15^ to reduce the risk of confounding factors when thresholds specific for ARMD were devised. In all such cases, the intraoperative findings at the revision and the results of histopathological and microbiological analyses confirmed the absence of ARMD.

The patients were divided into ARMD and non-ARMD groups. The ARMD group included patients who had undergone or were awaiting revision for ARMD, and those with cross-sectional imaging evidence of ARMD who were under surveillance because of their own or their clinician’s preference. Recommendations for revision surgery were always based on the outcome of the complete clinical assessment and never on the blood metal ion concentrations alone^13-15^. A diagnosis of ARMD was made if there was cross-sectional imaging and intraoperative evidence of a pseudotumor (a cystic, solid, or mixed mass communicating with the hip joint), or if there was substantial metallosis, synovitis, tissue damage and/or necrosis in the absence of a pseudotumor^4,30,32-35^. In patients undergoing revision, the diagnosis of ARMD was confirmed if there was also histopathological evidence of lymphocytic infiltrates (including aseptic lymphocytic vasculitis and associated lesions) and a phagocytic macrophage response to metal wear debris, with or without tissue necrosis^36-38^.

The non-ARMD group included all patients who did not undergo a revision for ARMD or who did not demonstrate cross-sectional imaging evidence of ARMD, regardless of symptoms^14,15^. Patients with bilateral BHRs who had ARMD in 1 or both hips were considered to have ARMD; otherwise, the patient was placed in the non-ARMD group. If patients had undergone multiple blood tests, the most recent result was used if the patient was in the non-ARMD group, with the result immediately prior to the revision used if the patient was in the ARMD group. For patients with evidence of ARMD on cross-sectional imaging, the most recent result was used.

### Statistical Analysis

Baseline differences between the patients in the ARMD group and those in the non-ARMD group were assessed using either the chi-square test (for sex) or 2-sided unpaired t tests (for age, femoral head size, and time to the blood test). Unpaired t tests were also used to compare the logarithms of the 3 blood metal ion parameters (cobalt, chromium, and the maximum value of either cobalt or chromium) between the ARMD and non-ARMD groups.

The use of receiver operating characteristic (ROC) curve analysis for assessing the ability of blood metal ion testing to identify patients with ARMD has been described^14,15^. Previously devised implant-specific blood metal ion thresholds (2.15 μg/L of cobalt for unilateral BHR, 5.5 μg/L for the maximum of either cobalt or chromium for bilateral BHR, and 3.57 μg/L of cobalt for unilateral Corail-Pinnacle implants)^14,15^ were applied to the validation cohort, with ROC curve analysis used to establish the discriminatory characteristics of the 3 respective thresholds for identifying patients with ARMD. The area under the curve (AUC), sensitivity, specificity, positive predictive value (PPV), negative predictive value (NPV), and positive and negative likelihood ratios were calculated with their respective 95% confidence intervals (CIs) for each implant (unilateral BHR, bilateral BHR, unilateral Corail-Pinnacle).

Fixed thresholds proposed by regulatory authorities in the U.K. (7 μg/L)^5^ and U.S. (3 μg/L and 10 μg/L)^18^ were applied to the validation cohort and compared, using the McNemar test, with the implant-specific thresholds^14,15^ in terms of the number of patients with ARMD who were not detected. A p value of <0.05 was considered significant for all analyses.

## Results

The validation cohort included 710 patients with a total of 803 MoM hip arthroplasties (Table I and Fig. 1), of which 75 (11%) were in the ARMD group and 635 (89%) were in the non-ARMD group. All blood metal ion parameters were significantly higher in the ARMD group than in the non-ARMD group (p < 0.001; Table II).

**TABLE I:** Patient Demographics (N = 803 Metal-on-Metal Hips in 710 Patients)*

Parameter	All Patients	ARMD Group	Non-ARMD Group	P Value: ARMD Versus Non-ARMD
Unilateral BHR				
No. (%) of patients	323 (100)	38 (12)	285 (88)	
Sex (F/M)	117/206	27/11	90/195	<0.0001‡
Age at blood test† *(yr)*	63.0 (32.5-84.4)	59.8 (34.3-77.5)	63.4 (32.5-84.4)	0.0673
Time between op. and blood test† *(yr)*	9.1 (1.9-15.2 )			
Femoral head size† *(mm)*	50.5 (38-58)	47.2 (38-58)	50.9 (38-58)	0.0001‡
Bilateral BHR				
No. (%) of patients	93 (100)	17 (18)	76 (82)	
Sex (F/M)	33/60	12/5	21/55	0.0022‡
Age at blood test† *(yr)*	63.5 (36.5-82.6)	59.3 (49.4-70.4)	64.4 (36.5-82.6)	0.0069‡
Time between op. and blood test† *(yr)*	8.2 (1.2-14.3 )			
Femoral head size† *(mm)*	50.7 (42-58)	48.4 (42-54)	51.0 (42-58)	0.0088‡
Unilateral Corail-Pinnacle				
No. (%) of patients	294 (100)	20 (7)	274 (93)	
Sex (F/M)	210/84	12/8	198/76	0.3599
Age at blood test† *(yr)*	78.7 (52.1-95.4)	74.0 (54.6-87.8)	79.1 (52.1-95.4)	0.0143‡
Time between op. and blood test† *(yr)*	3.6 (1.1-8.4 )			

* ARMD = adverse reactions to metal debris, BHR = Birmingham Hip Resurfacing, F = female, and M = male.

† The values are given as the mean with the range in parentheses.

‡ A significant difference.

**TABLE II:** Blood Metal Ion Concentrations*

Parameter	All Patients	ARMD Group	Non-ARMD Group	P Value: ARMD Versus Non-ARMD
Unilateral BHR				
No. (%) of patients	323 (100)	38 (12)	285 (88)	
Implant-specific threshold *(μg/L)*	2.15 (cobalt)			
Concentration† *(μg/L)*				
Cobalt	1.40 (1.00-1.95)	9.75 (2.35-31.1)	1.30 (1.00-1.70)	<0.0001‡
Chromium	1.29 (0.90-2.40)	8.92 (3.50-33.4)	1.18 (0.80-1.80)	<0.0001‡
Maximum of cobalt or chromium	1.50 (1.10-2.55)	10.5 (4.16-44.9)	1.40 (1.00-2.00)	<0.0001‡
Bilateral BHR				
No. (%) of patients	93 (100%)	17 (18%)	76 (82%)	
Implant-specific threshold *(μg/L)*	5.5 (maximum of cobalt or chromium)			
Concentration† *(μg/L)*				
Cobalt	2.10 (1.50-3.30)	6.30 (4.80-22.5)	1.80 (1.30-2.52)	0.0002‡
Chromium	2.30 (1.40-5.10)	8.50 (5.20-24.1)	2.04 (1.30-2.90)	0.0002‡
Maximum of cobalt or chromium	2.50 (1.70-5.10)	8.50 (5.36-24.1)	2.30 (1.60-2.92)	0.0001‡
Unilateral Corail-Pinnacle				
No. (%) of patients	294 (100%)	20 (7%)	274 (93%)	
Implant-specific threshold *(μg/L)*	3.57 (cobalt)			
Concentration† *(μg/L)*				
Cobalt	1.12 (0.65-2.75)	4.66 (1.50-8.07)	1.06 (0.65-2.46)	0.0008‡
Chromium	1.09 (0.83-1.77)	2.47 (1.75-4.16)	1.07 (0.78-1.66)	<0.0001‡
Maximum of cobalt or chromium	1.36 (0.88-2.87)	4.66 (2.17-8.07)	1.30 (0.88-2.49)	<0.0001‡

* ARMD = adverse reactions to metal debris, and BHR = Birmingham Hip Resurfacing.

† The values are given as the median with the interquartile range in parentheses.

‡ A significant difference.

### Validation of Implant-Specific Blood Metal Ion Thresholds (Table III)

*Unilateral BHR cohort (n = 323):* The AUC in the validation cohort was 89.4% (CI = 82.8% to 96.0%), compared with 90.5% (CI = 82.8% to 98.1%) in the derivation cohort^14^. The implant-specific unilateral BHR threshold (2.15 μg/L of cobalt) had a sensitivity (percentage of patients with ARMD who had a cobalt concentration above the threshold), specificity (percentage of patients without ARMD who had a cobalt concentration below the threshold), PPV (percentage of patients with a cobalt concentration above the threshold who had ARMD), and NPV (percentage of patients with a cobalt concentration below the threshold who did not have ARMD) of 78.9%, 86.7%, 44.1%, and 96.9%, respectively, for distinguishing between patients with and without ARMD after unilateral BHR in the validation cohort.

**TABLE III:** Results of Receiver Operator Characteristic Analysis*

									Likelihood Ratio†	
	Blood Metal Ion Threshold *(μg/L)*	AUC† *(%)*	Sensitivity† *(%)*	Specificity† *(%)*	PPV† *(%)*	NPV† *(%)*	Misclassification *(%)*	No. of Patients with ARMD Missed	Positive	Negative
Unilateral BHR (n = 323)	2.15 (cobalt)	89.4 (82.8-96.0)	78.9 (66.0-91.9)	86.7 (82.7-90.6)	44.1 (32.3-55.9)	96.9 (94.7-99.0)	14.2	8	5.92 (4.22-8.31)	0.24 (0.13-0.45)
Bilateral BHR (n = 93)	5.5 (maximum of cobalt or chromium)	89.2 (81.3-97.1)	70.6 (48.9-92.2)	86.8 (79.2-94.4)	54.5 (33.7-75.4)	93.0 (87.0-98.9)	16.1	5	5.37 (2.78-10.32)	0.34 (0.16-0.71)
Unilateral Corail-Pinnacle (n = 294)	3.57 (cobalt)	76.9 (63.9-90.0)	65.0 (44.1-85.9)	85.4 (81.2-89.6)	24.5 (12.9-36.1)	97.1 (95.0-99.2)	16.0	7	4.45 (2.90-6.85)	0.41 (0.23-0.75)

* ARMD = adverse reactions to metal debris, AUC = area under the curve, BHR = Birmingham Hip Resurfacing, NPV = negative predictive value, and PPV = positive predictive value.

† The 95% CI is given in parentheses.

*Bilateral BHR cohort (n = 93):* The AUC in the validation cohort was 89.2% (CI = 81.3% to 97.1%) compared with 91.0% (CI = 84.5% to 97.4%) in the derivation cohort^15^. Use of the implant-specific threshold for bilateral BHR (maximum value of either cobalt or chromium of 5.5 μg/L) resulted in a sensitivity, specificity, PPV, and NPV of 70.6%, 86.8%, 54.5%, and 93.0%, respectively, for distinguishing between patients with and without ARMD after bilateral BHR in the validation cohort.

*Unilateral Corail-Pinnacle cohort (n = 294):* The AUC in the validation cohort was 76.9% (CI = 63.9% to 90.0%) compared with 79.6% (CI = 68.8% to 90.4%) in the derivation cohort^14^. The implant-specific threshold for the unilateral Corail-Pinnacle implants (3.57 μg/L of cobalt) had a sensitivity, specificity, PPV, and NPV of 65.0%, 85.4%, 24.5%, and 97.1%, respectively, for distinguishing between patients with and without ARMD after unilateral Corail-Pinnacle implants in the validation cohort.

### Implant-Specific Thresholds Versus Fixed Thresholds Proposed by Regulatory Authorities

Using implant-specific thresholds, we missed 20 patients with ARMD (2.8% of the patients in our validation cohort). Significantly more patients with ARMD were missed when we used the fixed threshold of 7 μg/L (4.9%, n = 35; p = 0.0003) or 10 μg/L (6.5%, n = 46; p < 0.0001). Use of the fixed threshold of 3 μg/L resulted in 1 more patient with ARMD being missed (3.0%, n = 21) than when we used the implant-specific thresholds; however, this difference was not significant (p = 1.0).

## Discussion

Although blood metal ion levels are commonly measured during patient follow-up, there is presently no international consensus regarding the appropriate threshold(s) for clinical concern^5-7,18,39^. The authors of 2 recent studies reported the novel observation of implant-specific blood metal ion thresholds in patients treated with unilateral or bilateral BHR or unilateral Corail-Pinnacle implants^14,15^. These thresholds were effective for identifying patients at low risk of ARMD, with fewer patients with ARMD being missed than when the fixed thresholds proposed by regulatory authorities were used^5,18^. We have now validated the findings from these derivation studies^14,15^ in our large multicenter study.

The validation study was large—its size was similar to that of the derivation cohort—and it was truly external as it included patients from 3 geographically different centers^40,41^. Very similar findings were observed when the implant-specific thresholds for unilateral BHR, bilateral BHR, and unilateral Corail-Pinnacle implants^14,15^ were applied to the validation cohort, with the 3 thresholds demonstrating a good ability to distinguish between patients with and without ARMD (a high AUC) with good and balanced sensitivity and specificity. Furthermore, the implant-specific thresholds were effective for identifying hips without ARMD (a high NPV). These characteristics were generally better in the BHR validation cohorts than in the Corail-Pinnacle validation cohort, which parallels the findings in the derivation studies^14,15^. Although the implant-specific thresholds performed marginally better overall in the derivation cohort^14,15^ than in the validation cohort, this is to be expected. Statistical models invariably perform better in the derivation cohort than in external cohorts because they are intimately related to the patient, surgical, and unmeasured factors from the derivation study, which can never be reproducible externally^42,43^. However, the degree to which our findings in the validation cohort parallel those in the derivation studies is reassuring.

Using the implant-specific thresholds in our validation cohort, we missed significantly fewer patients with ARMD than we missed when using the fixed threshold of 7 μg/L proposed by the U.K. MHRA^5^ or the fixed U.S. threshold of 10 μg/L^18^; in fact, use of the 10 μg/L threshold more than doubled the number of patients with ARMD who were missed. When we applied the lower U.S. threshold (3 μg/L) to the validation cohort we missed 1 more patient with ARMD than when we used the implant-specific thresholds, but this difference was not statistically significant. However, as previously discussed^14,15^, such a difference is considered clinically relevant given the destructive potential of ARMD and poor outcomes reported following MoM hip revision surgery^44^.

It is recommended that similar implant-specific blood metal ion thresholds be developed for other commonly used MoM hip arthroplasty designs, as such thresholds may also perform better than the fixed thresholds currently recommended by regulatory authorities^5,18^. Although their methodology and selection criteria differed from those in the derivation studies^14,15^, Hart et al. observed that 7 μg/L was the “optimal” threshold for predicting failure of the recalled Articular Surface Replacement (ASR; DePuy) hip resurfacing and ASR XL total hip arthroplasty^13^. This finding provides further support for the belief that, similar to MoM hip arthroplasty revision rates^3,16^, clinically important blood metal ion thresholds are implant-specific. Authors of future studies should ensure that they apply the same methodology as described here and previously^14,15^, which includes focusing on ARMD-related failures (including those not treated with revision but identified on imaging), as we consider these methods largely responsible for the better diagnostic performance observed with the implant-specific thresholds compared with the blood metal ion thresholds from previous studies^9,12,13^.

The management of patients after MoM hip arthroplasty is complex^39^. As observed in this cohort (Table I) and previously^4,17,28^, there are recognized risk factors for developing ARMD such as female sex, young age at arthroplasty, and a small resurfacing femoral head. These factors are useful for risk-stratifying patients for surveillance^18,39^. It is important to recognize that no single investigation should be used alone in the clinical decision-making process for patients who have undergone MoM hip arthroplasty^18^. Therefore, blood metal ion levels should be only a part of the complete clinical assessment.

Although fixed thresholds proposed by worldwide regulatory authorities are widely used^5,18^, it is worth considering how they were formulated. In 2010, the MHRA first proposed 7 μg/L as the blood metal ion threshold for clinical concern, which was considered reasonable at the time given that ARMD was a new entity^5^. However, the MHRA still recommends that clinicians use the 7 μg/L threshold^5^ despite multiple studies suggesting evidence to the contrary, with lower thresholds being preferred^9-12,14,15^. The more recent consensus statement from experts in the U.S. represents an attempt to risk-stratify patients with MoM implants to assist in management, which is commendable^18^. However, the 3 μg/L and 10 μg/L thresholds that they recommended appear to have been arbitrarily selected rather than supported by clinical data^18^. In contrast, the implant-specific thresholds have now been assessed in nearly 1,500 patients with MoM hip implants from 4 different European centers^14,15^. Implant-specific thresholds were consistently shown, in both the derivation studies^5,18^ and our validation cohort, to be effective for identifying patients at low risk of ARMD after BHR or Corail-Pinnacle implants, and we missed fewer patients with ARMD than we missed when we used fixed thresholds proposed by regulatory authorities. Although the prevalence of ARMD varies among different institutions because of differences in surveillance methods and thresholds for revision^45^, this validation study has demonstrated that implant-specific blood metal ion thresholds perform well in different centers. We therefore consider our evidence-based implant-specific thresholds to be useful for managing patients after BHR and Corail-Pinnacle implants.

This study has limitations. In center 1, not all asymptomatic patients underwent blood metal ion sampling. This selection bias existed in the derivation cohorts^14,15^ and may affect the generalizability of implant-specific thresholds. However, universal blood sampling was performed at the other 2 validation centers, suggesting that implant-specific thresholds are effective in centers using either universal or targeted blood sampling. Another selection bias at all centers was the use of targeted cross-sectional imaging for asymptomatic patients with increased blood metal ion levels. Although this reflects the approach used in the clinical setting, where follow-up resources must be rationalized^5-7,12,14,15,17^, it is possible that some asymptomatic patients had silent ARMD but were incorrectly assigned to the non-ARMD group. The use of targeted imaging for asymptomatic patients therefore has the potential to affect the discriminatory characteristics reported in the validation cohort; however, a previous sensitivity analysis of patients undergoing cross-sectional imaging produced results similar to those for the whole cohort^15^. Although the validation cohort was large and its size was similar to that of the derivation cohort, the bilateral BHR group (n = 93) was smaller in the validation study than in the derivation study (n = 185)^15^. This may have affected the discriminatory characteristics in the bilateral BHR validation group. There were also limitations related to using only a single blood metal ion reading for each patient in the analysis, and the influence of patient and laboratory factors on blood metal ion levels have been detailed previously^14,15^. Finally the validated thresholds apply only to patients with a unilateral or bilateral BHR implant or a unilateral Corail-Pinnacle implant.

In conclusion, this large external multicenter validation study confirmed that patients with BHR or Corail-Pinnacle MoM implants and blood metal ion levels below newly devised implant-specific thresholds^14,15^ were at a low risk of ARMD. Using these implant-specific thresholds enabled us to miss fewer patients with ARMD than when we used current fixed thresholds proposed by regulatory authorities^5,18^. We therefore recommend using implant-specific blood metal ion thresholds when managing patients who have undergone MoM hip arthroplasty with BHR or Corail-Pinnacle implants.
